# Trust the Capitonnage in the Giant Cyst: Case Report

**DOI:** 10.1055/s-0038-1675370

**Published:** 2018-10-29

**Authors:** Murat Oncel, Huseyin Yildiran, Guven Sadi Sunam

**Affiliations:** 1 Department of Thoracic Surgery, Selcuk University Medical Faculty, Selcuk University, Konya, Turkey; 2 Department of Thoracic Surgery, Kars Harakani State Hospital, Kars, Turkey

**Keywords:** giant cyst hydatid, lung, surgery

## Abstract

Cyst hydatid in the lung is still a health problem for many countries. It develops in the lung and can grow into the lung parenchyma. When it is diagnosed as a giant cyst, surgery should be performed. In the surgery, capitonnage is necessary to protect the lung parenchyma.

Echinococcosis is an infestation caused by the *Echinococcus granulosus* and humans are the intermediate hosts. Many countries are endemic, especially the Middle East, South America, and eastern and southeastern region of Turkey. 1 The lung and the liver are the commonly involved organs. We aimed to present this interesting case report because of the age of the patient and the diameter of the cyst.

## Case Report

**Fig. 1:**
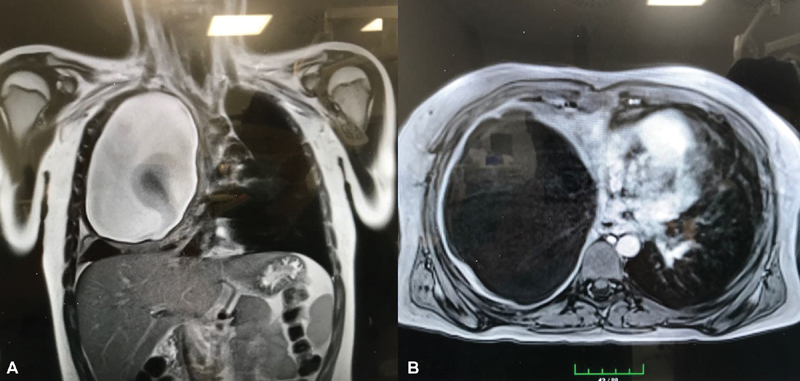
Magnetic resonance images of cystic lesion located in the right hemithorax, ( **A** ) coronal and ( **B** ) axial section. The germinative membrane is seen in the cyst fluid.

**Fig. 2:**
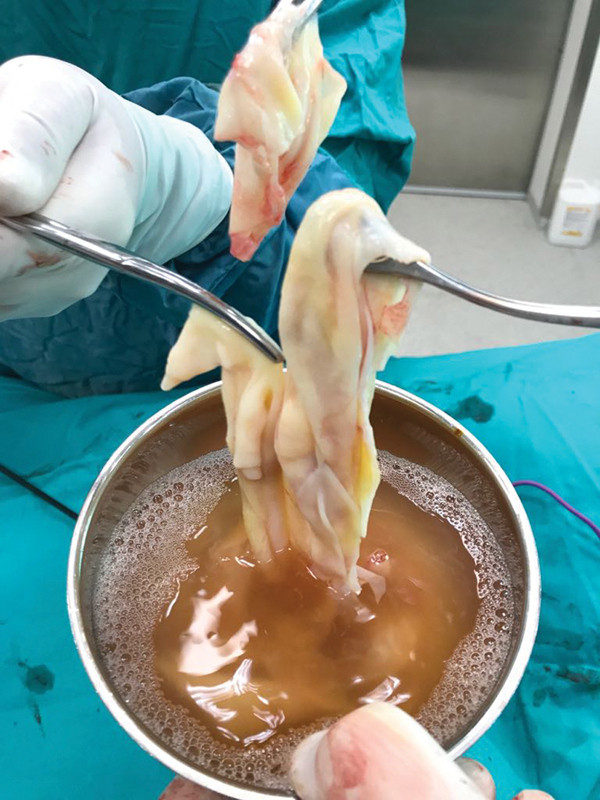
The germinative membrane of the giant cyst.

**Fig. 3:**
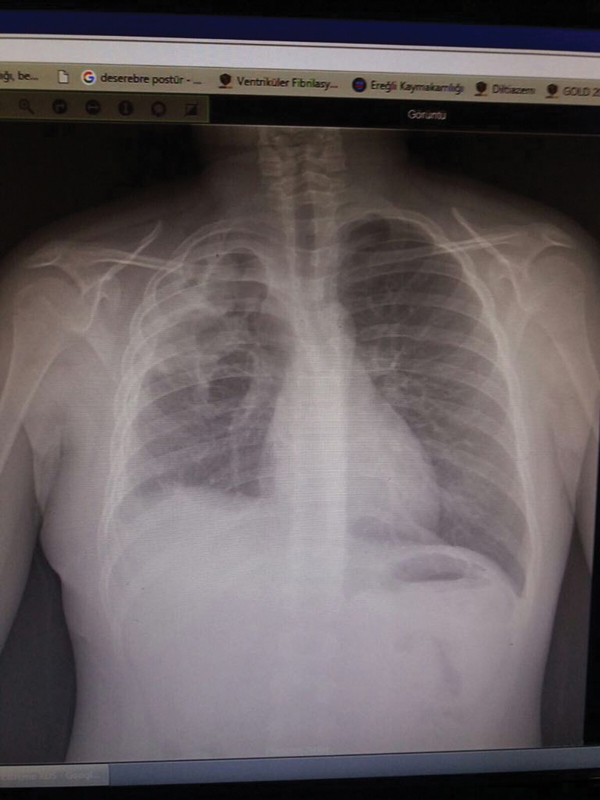
The postoperative chest X-ray showed the expanded right lung parenchyma.

A 12-year-old boy admitted to the hospital with right chest pain and shortness of breath. He had no other complaints such as cough, fever, or hemoptysis. During the examination, the right hemithorax respiratory sounds were diminished and the other side was normal. He had shortness of breath during exercise. On posteroanterior chest radiograph, there was a huge well-circumscribed opacity in the right hemithorax. Magnetic resonance showed a 15 × 16-cm cystic lesion which was filling the right hemithorax totally. Mediastinum and the heart had deviated to the left hemithorax ( Fig. 1 ). Surgery was planned immediately. In the surgery, thoracic exploration was done via single-port videothoracoscopy. First, utility incision was performed and then needle aspiration was done to aspirate the cystic fluid totally. After covering around the punctured place by a gauze with povidone iodine, the cyst wall was opened. The germinative membrane was removed in pieces, and the pouch of the cyst was checked for bronchial orifice. Then cystectomy and capitonnage were performed on the cyst located in the entire right upper lobe through utility thoracotomy at the level of 4th intercostal area ( Fig. 2 ). After air leak was controlled, the expansion of the right upper lobe was provided in the postoperative period ( Fig. 3 ).

## Discussion

Cyst hydatid is found in the form of eggs in the feces of carnivorous animals such as dogs, wolves, foxes, and jackals, and it enters the human system as an embrion. If the cyst manages to reach the lung, four scenarios may happen: (1) the parasite dies, the cyst is lost spontaneously, and the cuticle undergoes fibrosis; (2) cystic fluid is expectorated with a bronchial drainage; (3) the cyst dies because of infection between the cyst wall and cuticle, and the infection occurs in the cystic cavity; and (iv) the cyst continues to grow according to the compliance of the lung parenchyma. 2 3

Cysts may reach very large dimensions as the lungs have more elasticity. Lamy et al describes cyst over 4 cm as a giant cyst, while Halezeroglu et al and Karaoglanoglu et al define cyst over 10 cm as a giant cyst. 3 4 5 Giant hydatid cysts can be ruptured into the bronchial system or pleural cavity and they can be lead to some complications by putting pressure on the vital organs. They should be treated as soon as diagnosed. Surgery is the mode of treatment for giant lung cyst hydatid in children. Treatment with albendazole or mebendazole is preferred in patients who have noncomplicated small cysts and who do not tolerate or accept surgery. The surgical technique is not different between giant lung cyst hydatid and simple lung cyst hydatid. 3 4 Regardless of the cyst size, the lung parenchyma should be protected and lung resection should not be done as much as possible. Morbidity and mortality rates are very low in adult patients. 1 3 The morbidity rate is higher in the case of giant lung cyst. 4 6 Prolonged air leak (10–19%), empyema (7%), pneumonia, and sterile air gap are some of the reasons of morbidity. 6 However, giant lung hydatid cysts in children are more morbid as compared with adults, and it can be attributed to complications such as atelectasis and pneumonia which occur because of pain and unexpanded lung in short time. Both giant cyst incidence and lung resection rates in children are higher than adults. 7 8 In this case report, we performed capitonnage without parenchyma resection for a cyst which was located in the upper lobe. Also, the minimally invasive surgery with single port and utility incisions provided an excellent postoperative outcome. Lung expansion was completed in postoperative hour 2 and the hospital stay was without pain and respiratory problems.

As we added the giant lung hydatid cysts to our experience through video-assisted thoracoscopic surgery (VATS), morbidity and hospitalization time significantly decreased. We believe that VATS should be performed instead of thoracotomy as the latter induces atelectasis and pneumonia in hydatid cysts. 9 10 Capitonnage can be performed with VATS safely to protect the lung parenchyma.
